# Chronic lymphocytic leukemia with progressive anemia secondary to development of composite lymphoma

**DOI:** 10.1002/ccr3.2656

**Published:** 2020-01-10

**Authors:** Eric McGinnis, James T. England, Jeffrey W. Craig, Habib Moshref Razavi

**Affiliations:** ^1^ Department of Pathology and Laboratory Medicine University of British Columbia Vancouver BC Canada; ^2^ Division of Hematology University of British Columbia Vancouver BC Canada; ^3^ Department of Pathology and Centre for Lymphoid Cancer BC Cancer Vancouver BC Canada; ^4^ Fraser Health Authority Royal Columbian Hospital New Westminster BC Canada

**Keywords:** chronic lymphocytic leukemia, composite lymphoma

## Abstract

Deterioration of hematologic parameters in lymphoma patients is often attributed to disease progression, comorbidities, or treatment effects. Second primary malignancies occur at increased frequency in CLL and must also be considered.

## Graphical abstract

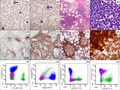

## 1 INTRODUCTION

A 73‐year‐old woman with a long‐standing history of untreated chronic lymphocytic leukemia (CLL) presented with fatigue and progressive anemia. A bone marrow biopsy was performed for suspicion of disease progression and exclusion of alternative conditions.

**Figure 1:**
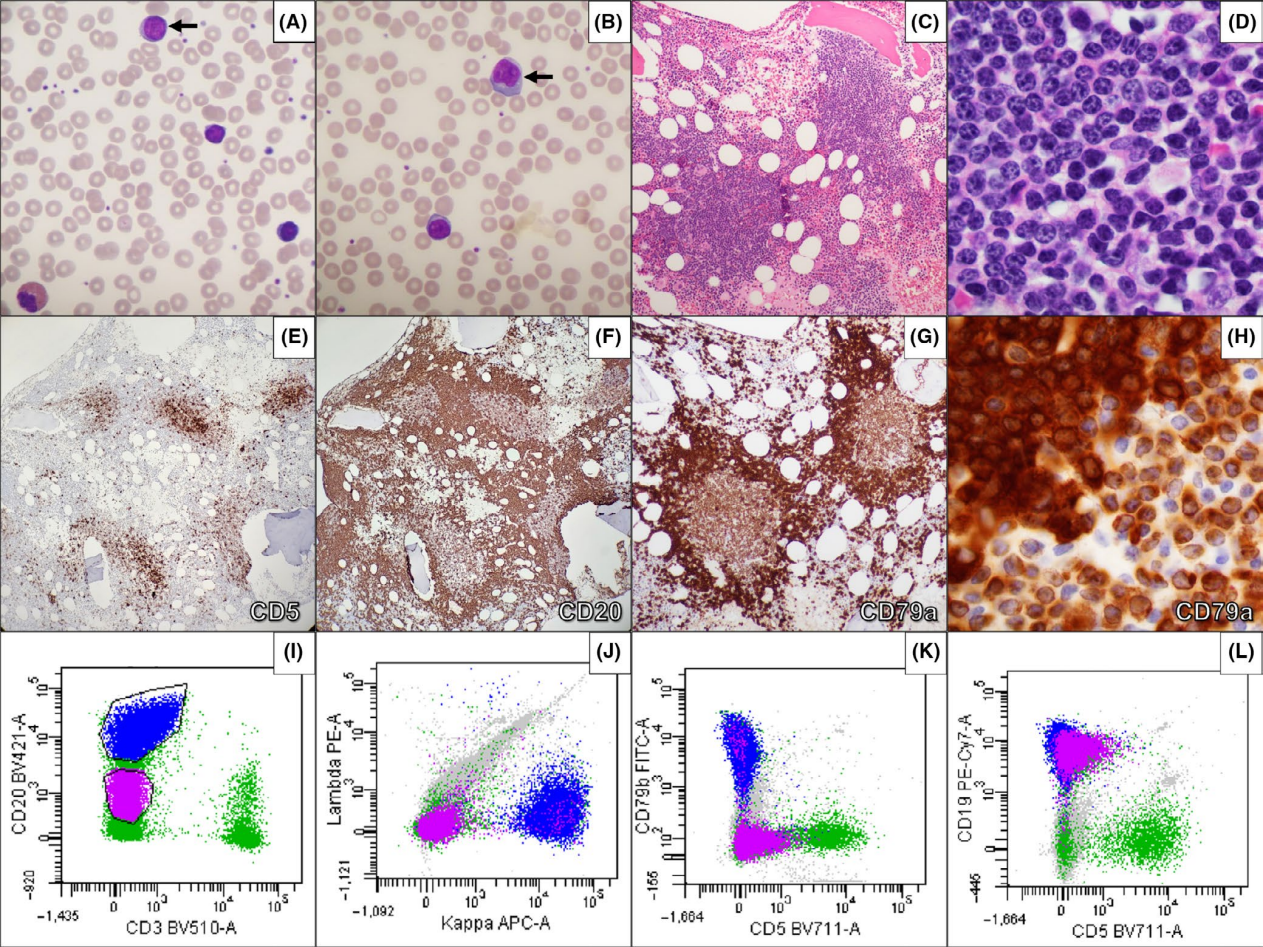
Peripheral blood film (A‐B, 60× objective) with dimorphic atypical lymphocytes and bone marrow biopsy (C, 10× objective; D, 100× objective) with nodular lymphoid infiltrates accounting for the majority of marrow cellularity. Two distinct B‐cell populations differentially express CD5, CD20, CD79, and sIg by immunohistochemistry (E‐F, 4× objective; G, 10× objective; H, 100× objective) and/or flow cytometry (I‐L, magenta: CLL; blue: second primary non‐Hodgkin lymphoma)

Her peripheral blood showed atypical lymphocytes with dimorphic cytologic features (Figure 1, panels A‐B) including a subset of intermediate‐sized cells with expanded cytoplasm (arrows). Her bone marrow was involved by nodular aggregates (Figure 1, panel C) of composite lymphoma consisting of two morphologically and immunophenotypically distinct populations of abnormal B lymphocytes: small mature cells (Figure 1, panel D, lower right) consistent with her known CLL and a surrounding population of larger cells with more open chromatin and distinct nucleoli (Figure 1, panel D, upper left) and antigen expression most suggestive of marginal zone or lymphoplasmacytic lymphoma.

The risk of developing a second primary malignancy is more than twice as high in patients with CLL than those without and must be considered in the setting of emerging cytopenias.^1^ Among secondary hematologic malignancies, most of this excess risk is due to additional non‐Hodgkin lymphomas.^2^ In this case, the patient's worsening anemia was attributed to the combination of her new secondary non‐Hodgkin lymphoma in composite with her known CLL.

## CONFLICT OF INTEREST

The authors have no conflicts of interest to disclose.

## AUTHOR CONTRIBUTIONS

EM: captured images and prepared the manuscript. JTE, JWC, and HMR: analyzed the case, provided images, and participated in preparation and revision of the manuscript.
